# Investigating the Fluid–Solid Interaction of Acid Nonionic Nanoemulsion with Carbonate Porous Media

**DOI:** 10.3390/molecules25061475

**Published:** 2020-03-24

**Authors:** Cláudio Regis dos Santos Lucas, Yanne Katiussy Pereira Gurgel Aum, Edson de Andrade Araújo, Tereza Neuma de Castro Dantas, Elayne Andrade Araújo, Talles Nóbrega Sousa, Pedro Tupã Pandava Aum

**Affiliations:** 1 Petroleum Engineering Faculty, Campus Salinópolis, Federal University of Pará—UFPA, Salinópolis 68721-000, Brazil; claudiolucaseq@yahoo.com.br (C.R.d.S.L.); edsonandradesp@gmail.com (E.d.A.A.); 2 Petroleum Science and Engineering Postgraduate Program, Federal University of Rio Grande do Norte—UFRN, Natal 59075-000, Brazil; 3 Chemical Engineering Department, Federal University of Amazonas—UFAM, Manaus 69080-900, Brazil; yanne@ufam.edu.br; 4 Chemical Engineering Department, Federal University of Rio Grande do Norte—UFRN, Natal 59075-000, Brazil; terezaneuma1011@yahoo.com.br (T.N.d.C.D.); elaynea_@hotmail.com (E.A.A.); talles22@hotmail.com (T.N.S.)

**Keywords:** O/W acid nanoemulsion, carbonate porous-media, retarded reaction, microCT imaging

## Abstract

The subject of rock–fluid interaction is important in cases where flow through porous media is occurring. One special case is when the fluid reacts with the porous matrix. In this case, the mass transfer and reaction rate control the dissolution pattern. This article aimed to study the interaction between an acid nanoemulsion system and a carbonate porous media. Nanoemulsions were developed to retard the rock’s dissolution and to promote the formation of conductivity channels. Nanoemulsions were prepared using ALK100 (alkyl alcohol ethoxylate) and RNX110 (alkylphenol ethoxylate) (nonionic surfactants), sec-butanol (co-surfactant), xylene isomers (oil phase), and a solution of HCl (aqueous phase). The obtained systems were characterized in terms of surface tension, droplet diameter, and reactivity. X-ray fluorescence/diffraction (XRF/XRD) and X-ray microtomography (microCT) were performed on carbonate porous media samples treated with the acid systems in order to observe the effects of the fluid–rock interaction. The results showed that the acid nanoemulsion, presenting a low oil content formulation, showed the low surface tension and droplet size characteristic of nanoemulsions. It was experimentally verified that the reactivity in the nanoemulsion media was mass-transfer-retarded, and that the wormhole pattern was verified under the studied conditions.

## 1. Introduction

Flow in porous media involves several phenomena and processes. When a fluid reacts with the porous matrix, the flow is then called reactive flow in a porous medium. In this case, the porous matrix is partially consumed and the dissolution pattern is strongly dependent on the fluid–rock interaction, comprising reactive species transport and the rate of reaction. Acidizing is an important application of this type of phenomenon, used in the petroleum industry. It typically involves the injection of hydrochloric acid [1] into a reservoir near the wellbore region under a fracture to form pressure. The acid flows through the porous medium, dissolving the matrix and forming flow channels called “wormholes” [2]. These channels are responsible for increasing permeability and thus reducing the loss of the flow energy due to damage or poor connection between the well and reservoir, thereby improving the production index [3]. Stimulation by acidizing is widely applied to carbonate formations, enhancing the production of a well. Carbonate rock formations play an important role in the global context once this type of rock contains nearly 50% of the estimated oil and gas resources in the world. 

A challenging task in carbonate acidizing is to control the extremely rapid acid–rock reaction. In other words, consumption of the entire volume of acid near the site of injection must be prevented to ensure that the treatment can reach a greater radial distance from the well and thus favor a more effective flow [4,5].

When the reaction is rapid, the mechanisms of diffusion and convection/advection drive the acid consumption in the liquid phase [6]. Controlling the reaction is of particular importance in formations with low permeability because the operational window has a limitation that forces it to work with low flows, thereby promoting prolonged contact between the acid and the formation. Retarded systems hinder acid diffusion or create barriers between the acid and the carbonate formation.

The most widely used retarded systems include gelled acids and emulsified acids (macroemulsions) [7,8]. The application of gelled acids is limited to scenarios involving low pumping pressure owing to the increased loss of the energy necessary for the effective displacement of highly viscous fluid.

Macroemulsion systems contain micelles of aqueous acid dispersed in a continuous oil phase. The function of the micelles in conventional emulsion systems is to trap the acid. As a consequence, it becomes difficult for acid to reach the reaction’s surface [9].

Studies reported in the literature have investigated the use of microemulsions as alternative retarded acid systems [8,10,11,12,13,14]. Microemulsions are stable thermodynamic systems formed by surfactants, co-surfactants, an oil phase, and the water phase. Such systems are optically transparent and characterized by large interfacial areas, low interfacial tension, and low viscosity [15,16,17,18]. Although the use of microemulsions is promising, their formation, in general, requires high concentrations of surfactants, which reduce their cost-effectiveness as retarded acid systems.

Nanoemulsions are potential alternatives to microemulsions and can exhibit similar properties at low surfactant concentrations [19,20,21,22,23]. A nanoemulsion is a dispersion of small droplets of two immiscible fluids, typically oil in water (O/W) or water in oil (W/O), with a mean droplet size of < 200 nm [24,25,26], requiring energy input for formation [27,28,29]. Microemulsions and nanoemulsions have a similar range of size diameters (< 200 nm), causing confusion in the definition. However, as the main difference, microemulsions are formed spontaneously. Therefore, a microemulsion does not need additional energy to be formed, in contrast to nanoemulsions. Another difference is related to stability. Microemulsions are thermodynamically stable. Thus, microemulsions could be stable for an undefined period; nanoemulsions are kinetically stable, which means that the droplet coalescence process is slow, but at a certain moment, the nanoemulsion can collapse. All these characteristics are related to the amount of surfactant available to form the micellar structures. In microemulsions, the higher concentration of surfactant favors a state of thermodynamic stability [28]. 

Several methods are used to obtain nanoemulsions. These methods are commonly categorized into high-energy and low-energy methods. High-energy methods involve high-energy homogenization techniques for dispersion and stabilization of the droplets to produce the nanoemulsions. Low-energy or low-intensity methods involve the generation of small droplets owing to changes in the surfactant–oil–water composition or changes in environmental conditions such as temperature or pressure [29,30].

The low-energy methods use the energy from the physicochemical process rather than mechanical forces applied to the system. Due to the cost reduction and ease of application, this route for nanoemulsion preparation is gaining attention [31,32]. 

The development of colloidal reactive systems such as emulsions, microemulsions, and nanoemulsions aims to obtain systems that can control acid–rock reactions. In acidizing, this delay in the reaction prevents face dissolution, increasing the efficiency in the formation of conductivity channels known as wormholes. In wellbore cementing, aqueous acid solutions are also used, and a high reaction rate can damage the cement sheath and can cause well instability problems. In this context, industrial research has focused on the development of colloidal systems for use in acidizing fluids. However, few studies have investigated the fluid–rock interactions between nanoemulsion and carbonate rock formations. 

Reaction in porous media are complex, and involve advection, diffusion, and reaction mechanisms occurring under capillary forces. Generally, the use of dimensionless parameters like Péclet and Damköhler numbers help to better understand and quantify the phenomena during the reactive flow process. The reactive flow dissolves the rock matrix, increasing porosity and permeability [33].

Evaluation of the change in porosity and permeability in carbonate rocks is usually carried out using computerized microtomography (microCT) techniques. From a three-dimensional image, it is possible to obtain both the structure of the porous matrix and the pattern of dissolution of the rock [34]. In this work, we were interested in proving the effectiveness of nanoemulsion systems to form wormhole patterns; therefore, we used microCT to visualize the structure formed. Another aspect generally not covered in the literature is how the acid reaction modifies and releases the surface. We were especially interested in morphological changes promoted by the acid–fluid rock interaction and whether it was possible through surface inspection to observe, at pore-scale, phenomena like face dissolution and to perceive the effect of the retarded acid systems.

Therefore, in this study, the fluid–rock interaction between an aqueous acid nanoemulsion system (ANS) and a carbonate formation was investigated. In the experiment, acid nanoemulsions were prepared using a low-energy method, and their capacities to retard the acid–carbonate reaction and dissolve carbonate formations were evaluated. Characterization was also done of the fluid, rock, and the fluid–rock interaction.

## 2. Results

### 2.1. Coreflood Experiments and X-Ray MicroCT Imaging

Table 1 summarizes the coreflood experiment results, where K_before_ represents the permeability of the core plug before the acid treatment. PVBT (pore volume to breakthrough) represents the pore volume necessary to reduce the differential pressure drop across the core near to zero, i.e., the volume of acid necessary to promote the wormhole channel breakthrough the core.

**Figure 1:**
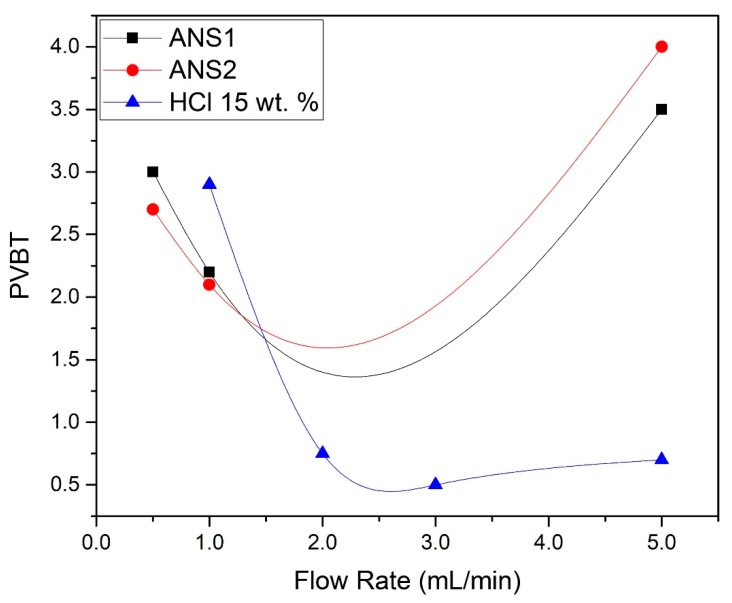
PVBT (pore volume to breakthrough) for ANS1, ANS2, and HCl 15.0 wt. %.

The results of the experiments showed that for the flow rate studied, the breakthrough volume was at minimum 1 mL/min, with PVBT = 2.2 and PVBT = 2.1 for the nanoemulsions ANS1 and ANS2, respectively. For both systems, the maximum PVBT was 5 mL/min. Figure 1 presents the plots of the PVBT. Additionally, the PVBT values were plotted for systems of 15 wt. % aqueous HCl at the same flow rates.

Analyzing Figure 1, it was concluded that near the range of 0.5 to 1 mL/min, the volume to breakthrough was lower than for aqueous HCl with the same concentration. This was further indication that the obtained systems retarded the dissolution reaction of the calcium carbonate.

**Figure 2:**
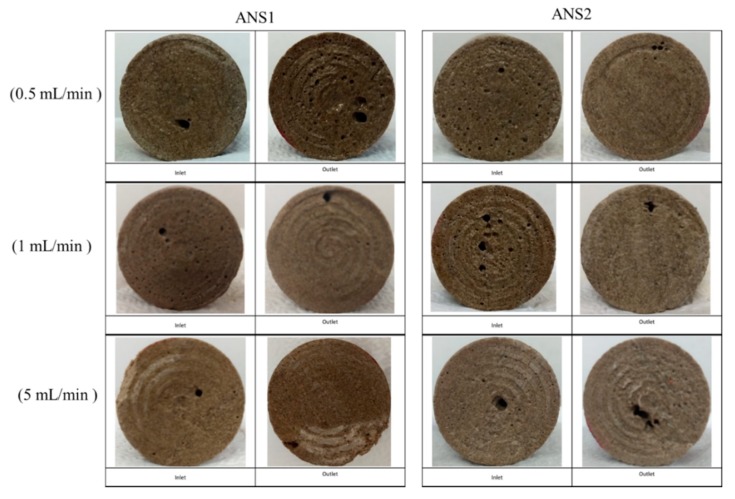
Inlet and outlet core plugs for the systems ANS1 and ANS2 at different flow rates.

To verify the wormhole formation in the cores, X-ray microCT scans were performed for the plugs IL-71D and IL-76D. These plugs were chosen because the plugs were acidified at lower flow rates. Figure 2 shows photographs of the core faces. 

No face dissolution was noticed in the core inlet faces for all experiments performed with the nanoemulsion systems. Figure 2 displays small cavities in the end faces, indicating wormhole channel formation. Low flow rates are critical once the acid spends more time interacting with the rock formation; in this condition, reaction control is essential to promote the wormhole pattern. Thus, the core plug imaging results indicated that the nanodroplets were effective to control the acid–rock reaction and ensure wormhole channel formation without face dissolution of the rock.

**Figure 3:**
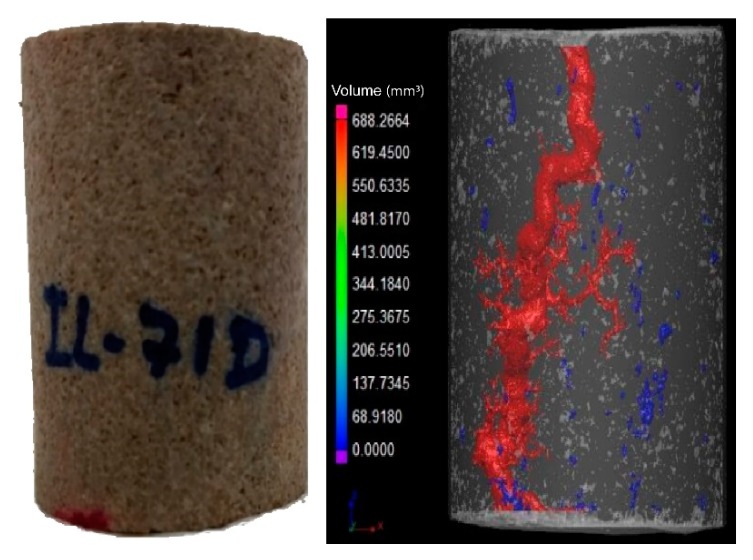
Imaging of wormhole structure using CT scan; ANS1; 0.5 mL/min (IL-71D).

**Figure 4:**
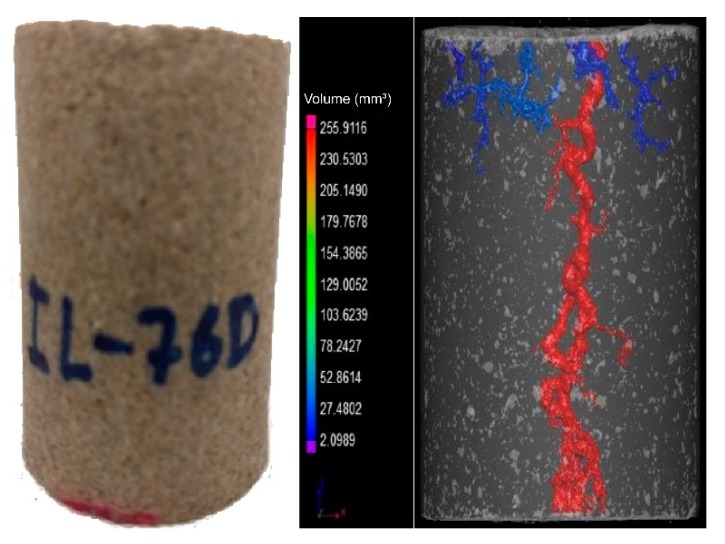
Imaging of wormhole structure using CT scan; ANS2; 0.5 mL/min (IL-76D).

It was observed that breakthrough was achieved for all experiments. In the outlet of the core, more than one point of breakthrough was noted, indicating a ramified wormhole structure. Figure 3 and Figure 4 show the core plug scans obtained by X-ray microCT after the core acid treatment.

**Figure 5:**
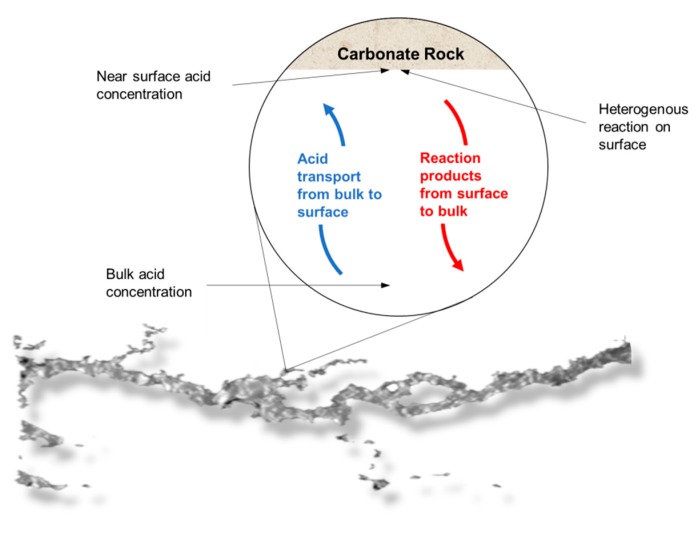
Representation of the heterogeneous reaction mechanism in a wormhole.

It was observed that for different flow conditions, the volume necessary to form the conductive channel changed. According to the data, the IL-71D acidized core showed wormholes with an average radius of 4.44 ± 7.95 mm, and IL-76D showed ones with 6.30 ± 7.34 mm. It was also observed that the reaction was retarded using the nanoemulsions. These effects arose once the reaction between the acid and the mineral occurred as a complex combination of the mass transfer of the acid to the surface of the rock and subsequent reaction on that surface, as illustrated in Figure 5. This type of reaction occurs at an interface and is called heterogeneous. The transport of the acid to the surface is convective, resulting from the mixture (advective) and the concentration gradient (diffusive). Since this are a complex effect, both contribute to the overall rate of reaction, which in fact determines the speed of the reaction; however, in many situations, one effect is greater than the other, allowing the other to be ignored.

### 2.2. Acid Nanoemulsion Preparation and Characterization

The first step to determining the nanoemulsion composition was to study the effect of the acid dilution in a predefined microemulsion. The dilution meant that at particular point, the surface tension increased sharply. It was not possible to separate the microemulsion from the nanoemulsion region through visual inspection of the point in ternary diagrams. 

**Figure 6:**
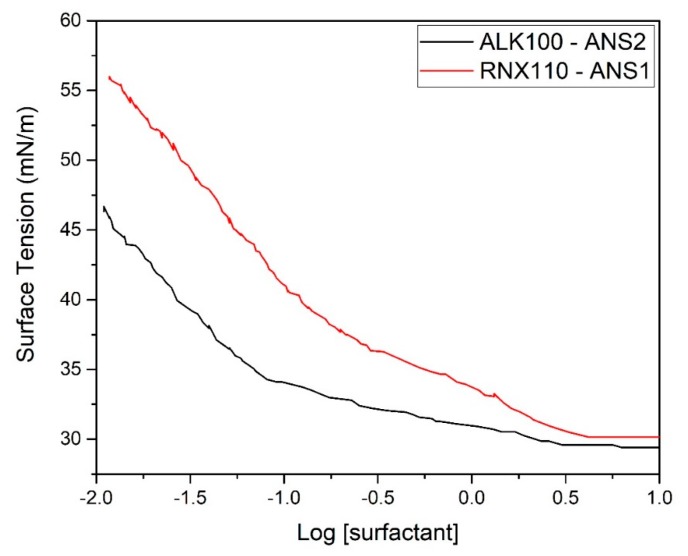
Plots of surface tension versus surfactant concentration.

However, it is known that in low surfactant concentrations, there is not enough active material to form microemulsions, so we verified the formation of nanoemulsions through the analysis of surface tension. Figure 6 shows the effect of dilution on surface tension.

From this graph, it can be observed that for both systems studied, at near 1 wt. % of the surfactant (LOG[RNX110] = 0), the surface tension suddenly increased. This behavior was an indication of the stability loss and signaled the limit of the critical micellar concentration.

To obtain nanoemulsions, we used the inversion phase method promoted by dilution of a binary mixture composed of surfactant, co-surfactant, and oil [19]. After the first aqueous droplet, the binary mixture become a water-in-oil microemulsion. Thus, the continuous addition of the water phase moved the point in the water vertex direction. Therefore, combining the results obtained in Figure 6, we started the dilution in the binary mixture and proceeded with the dilution of microemulsion system until 5% surfactant was achieved, above the limit determined in the surface tension study.

Based on this result, the nanoemulsions selected had a composition of 5 wt. % surfactants (S), at this concentration, one observe high stability and low surface tension, 2.5 wt. % cosurfactant (C), 91.5 wt. % aqueous solution (AP) of HCl 16.4 wt. %, and 1 wt. % oil phase (OP). An aqueous solution of HCl 15.0 wt. % was used to compare the acidizing performance. Table 2 summarizes the compositions of the reactive systems used.

The HCl concentration in the aqueous phase was varied in order to maintain the total concentration of HCl with the same dissolution power as that of aqueous HCl 15 wt. %.

**Figure 7:**
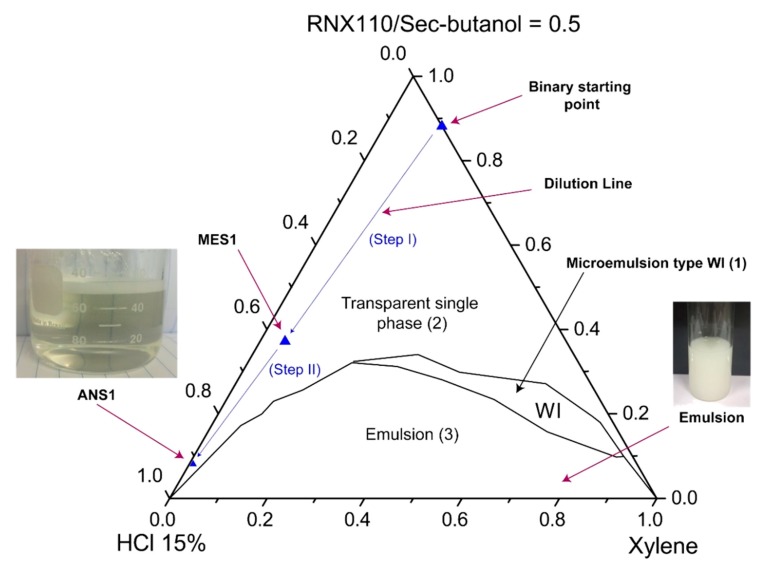
Phase diagrams for systems with RNX110.

**Figure 8:**
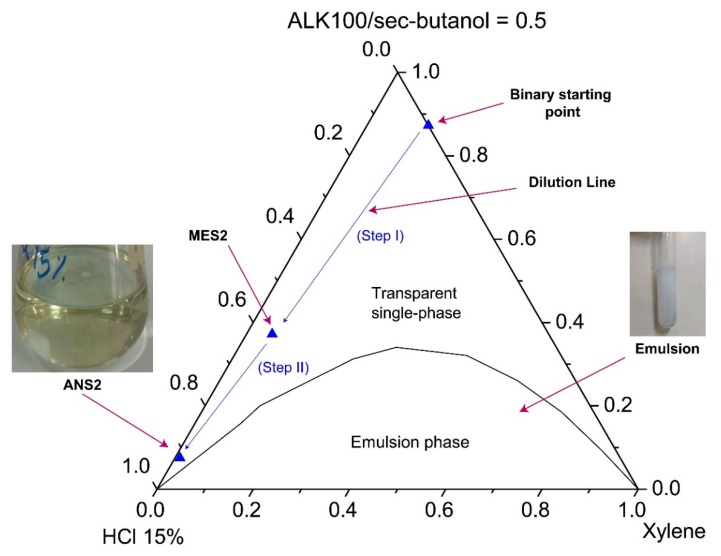
Phase diagrams for systems with ALK100.

The systems were characterized through visual inspection into (1) microemulsion type Winsor I, (2) emulsion, and (3) single transparent phase. The systems (1) of microemulsion type WI consisted of a two-phase system in which a microemulsion phase coexisted in equilibrium with an oil phase. We defined as emulsion the point at which the formulation became visually milky. A single transparent phase revealed the microemulsion (WIV) or nanoemulsion formation as a monophasic system. Figure 7 and Figure 8 show pseudoternary diagrams obtained for the two surfactants studied. From the diagrams, it was possible to observe three defined regions: (1) emulsion, (2) Winsor I (WI), and (3) a single transparent phase of microemulsion (WIV).

From the diagrams (Figure 7 and Figure 8), it was also possible to observe the composition of the points of the prepared nanoemulsion systems (ANS1 and ANS2). The first arrow (step I) represents the dilution of a pseudo-binary mixture (co-surfactant/surfactant and xylene) to form the intermediate microemulsion systems MES1 and MES2. The dilution process continued until the inversion point was reached and a nanoemulsion system was formed. The phase transition promoted by the water titration shifted the point composition to near the water vertex. The dilution process continued until a surfactant concentration of 5% was reached. The points of this composition were called ANS1 and ANS2. The presented methodology could be eventually scaled up to a field scenario (e.g., stimulation vessel), maintaining the sequence of first prepared water-in-oil (W/O) fluid with the additives, and then performing the dilution to promote the phase transition.

**Figure 9:**
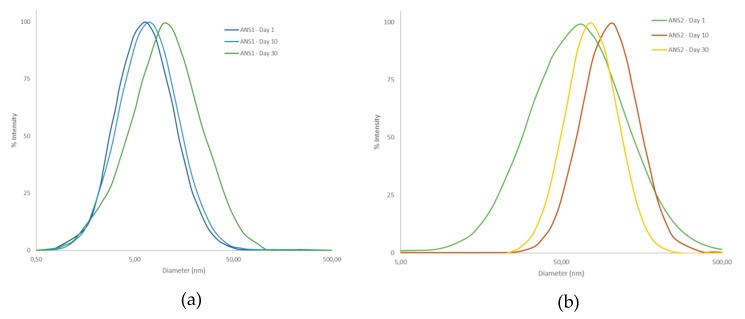
Particle size distribution curves of the systems (**a**) ANS1 and (**b**) ANS2.

Figure 9 shows the mean diameter for the acid nanoemulsion systems obtained. The system prepared with the surfactant RNX110 exhibited a larger particle diameter of around 80 nm, whereas the system prepared with ALK100 exhibited a particle diameter of around 60 nm. It was also observed that the particle diameter changed by less than 3% even after 30 days, indicating the high stability of the nanoemulsions prepared.

**Figure 10:**
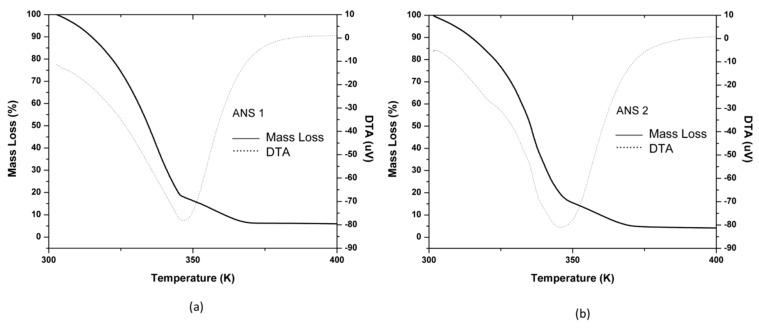
Thermogravimetric analysis (TGA-DTA) for system ANS1 (**a**) and ANS2 (**b**).

Thermogravimetric analysis (TGA-DTA) results for the systems ANS1 and ANS2 are shown in Figure 10a,b. The obtained systems presented similar mass loss steps between 300.15 K (27 °C) −367.15 K (94 °C). The mass retention rate considering a reservoir temperature of 328.15 K (55 °C) was 75%. The behavior difference observed after 367.15 K (94 °C) was due to each surfactant thermal resistance since, at this point, they presented the remaining mass approximately to theoretical surfactant quantity (5 wt. %).

These results were satisfactory and led to the conclusion that both systems could be used in a petroleum reservoir with a trivial temperature condition.

### 2.3. Porous Media Characterization

Indiana limestone was used as the porous medium to evaluate the reaction between the acid system and carbonate rock. The elementary composition and mineralogical analysis were done via XRF and XRD analyses applied to Indiana rock sample pulverized and sieved to a 200 mesh size. The identification of the elementary composition of major and trace elements was performed through FRX, where the sample was analyzed by Rh radiation between 15 and 50 kV. The results are shown in Table 3, where it is possible to observe that Indiana limestone is mostly composed of calcium, followed by other elements in much smaller quantities. 

**Figure 11:**
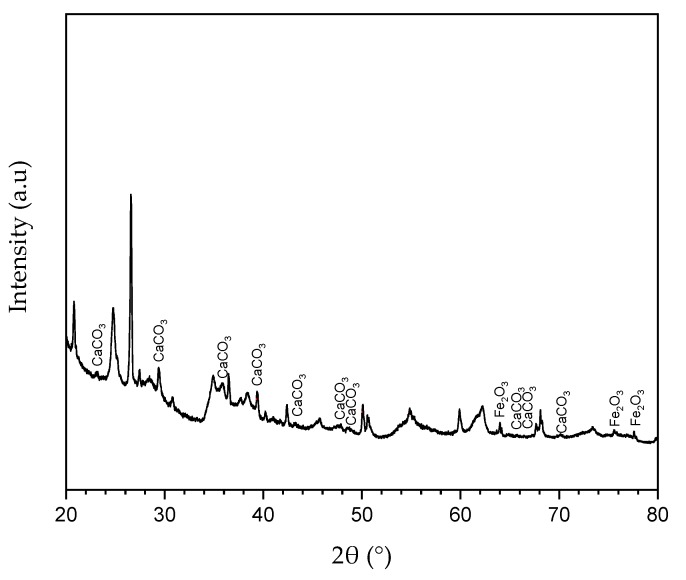
X-ray diffraction for Indiana limestone sample.

The mineralogical analysis was done via XRD by scanning the same sample from 0° to 160° (3°/min) with Kα (Cu) radiation at 40 kV and 20 mA. It was possible to observe through the associated peaks in Figure 11 that the sample consisted largely of calcium carbonate, followed by iron oxide and insignificant amounts of other compounds.

The porosity and the gas permeability for the samples were also determined, obtaining an average porosity near 11% and 140 mD of permeability. Core samples of 3.8 cm length and 5.0 cm diameter were analyzed. The porosity was determined based on Boyle–Marriote law, and the gas permeability by adjusting the curve flowrate versus pressure drop across the core.

### 2.4. Retarding Effect on Acid Calcium Carbonate Dissolution

**Figure 12:**
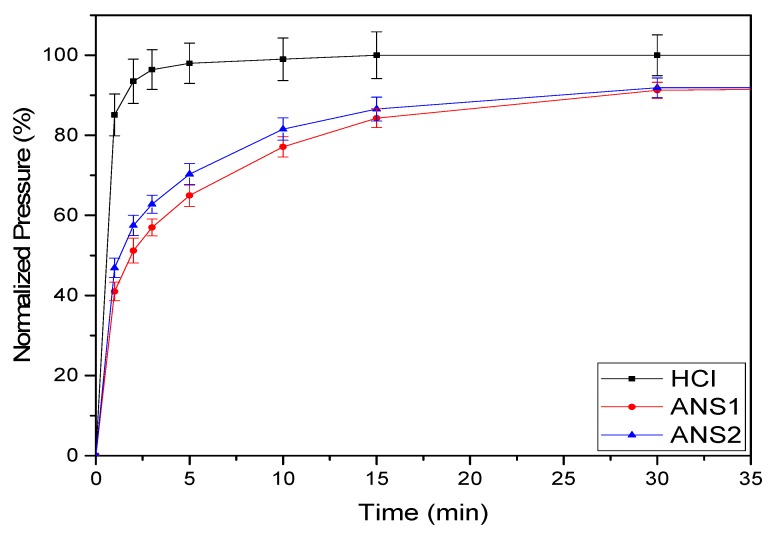
Consumption of calcium carbonate with time.

To calculate the consumption of calcium carbonate over time (η), measured the internal pressure in the reactor owing to the CO_2_ released as a byproduct of the dissolution reaction was measured as a function of time. Figure 12 shows the curves of calcium carbonate consumption over time (η) for each acid medium. For the HCl system, the curve rose sharply to 90% conversion and then continued to rise gradually until the maximum conversion. The results of the experiments indicated that the nanoemulsion systems promoted a significant reduction in the reaction rate, reaching 91% conversion at 30 min for both systems.

The curve slopes’ smoothing for the systems ANS1 and ANS2, shown in Figure 12, demonstrated that the calcium carbonate consumption was retarded in the microemulsion media.

**Figure 13:**
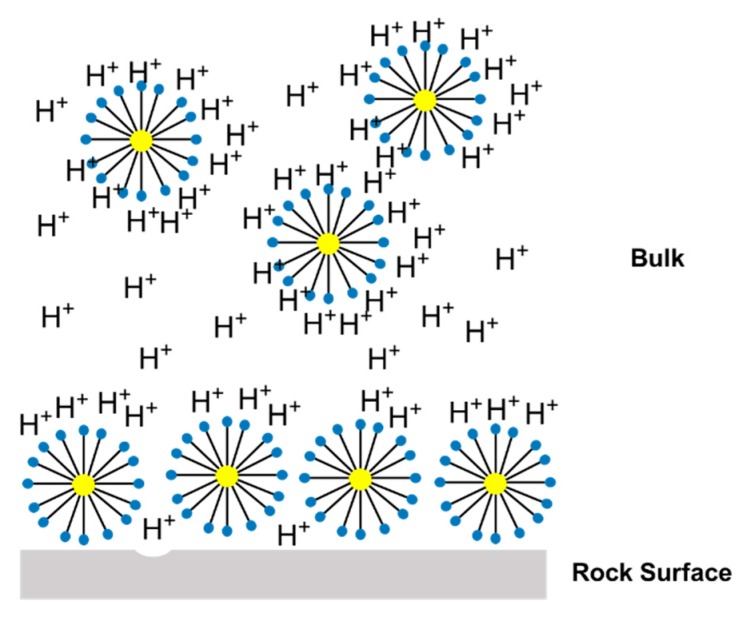
Representation of the acid oil-in-water nanoemulsion.

Figure 13 shows a representation of the acid nanoemulsion media; the three main factors that affect the reaction in nanoemulsion media are illustrated in this figure. The first factor is the interaction between the surfactant and the acid once the surfactants have available electrons in the ethoxylate molecules. This leads to a competition between the reaction and the carbonate rock. The second factor is the presence of micelles in the aqueous media, which affects the acid diffusivity and increases the resistance to acid diffusion. The third factor is the interaction between the micellar structures and the rock surface, which promotes the formation of a protective film and retards the reaction. All these factors contribute to retardation of the reaction between the acid and the calcium carbonate.

Nanoemulsion systems prepared with RNX110 as the surfactant promoted the maximum retardation of the reaction. This behavior can be explained by the fact that once the polar structure of RNX110 is formed by an aromatic, it promotes more significant attraction for hydrogen. Further, it was observed that the reaction for both nanoemulsion systems stopped near η = 91% of conversion. This behavior was a consequence of the acid trapped in the nanoemulsion medium and the interactions of the acid within the micellar structure, which prevented acid diffusion.

### 2.5. Wettability Surface Change after Acidizing

Wettability is an important parameter in an environment under capillary forces like a porous medium. The main objective of this study was to understand the affinity that the acid solutions had to the rock and how the rock surface was released after acidizing. The study of wettability alteration was carried out using Indiana limestone slices treated with the systems presented in Table 4.

**Figure 14:**
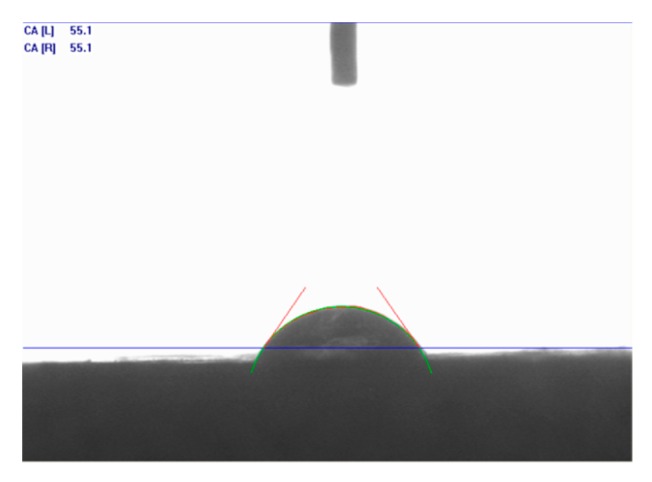
Contact angle for DW (distilled water) and Indiana limestone (55.1°).

**Figure 15:**
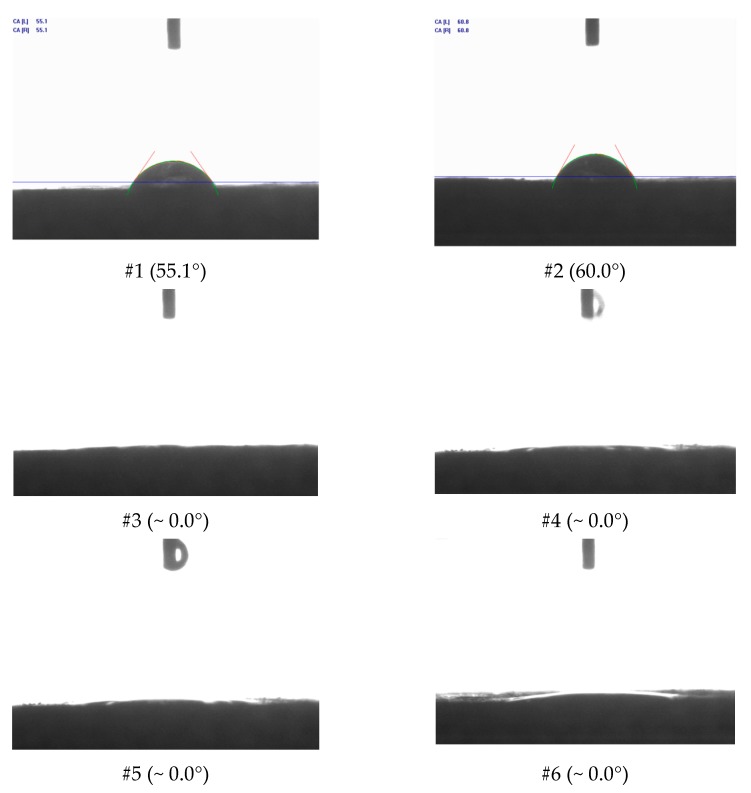
Contact angle in Indiana limestone measured with DW ( distilled water) after surface treatment with SA-1.54 wt. % HCl (#1), SA-15.00 wt. % HCl (#2), SSA-1.54 wt. % HCl (#3), SSA-15.00 wt. % HCl (#4), ANS1-1.54 wt. % HCl (#5) and ANS1-15.00 wt. % HCl (#6).

The contact angles were measured and photographs were taken using scanning electron microscopy (SEM) before and after treatment. Figure 14 shows the contact angle of distilled water (DW) (no treatment) in the Indiana limestone, and the data for the acid systems, taken immediately after counting the drop with the substrate, can be found in Figure 15.

As shown in Figure 14, it was observed that Indiana limestone was oil-wettable and that the presence of surfactant was decisive for the intense increase in the contact angle to the point that it was not possible to measure it, even after repeating the process several times. Therefore, as shown in Figure 15, it was not possible to compare the effect of increasing the concentration of HCl or the change in structure of the auto-associative system, limiting the analysis to stating that all systems studied containing the surfactant ALK100 were completely wettable in the Indiana limestone. In the case of the acid solution without surfactant, the increase in the acid concentration did not significantly change the contact angle. This behavior is important in considering how the rock surface is released after the acidizing process.

**Figure 16:**
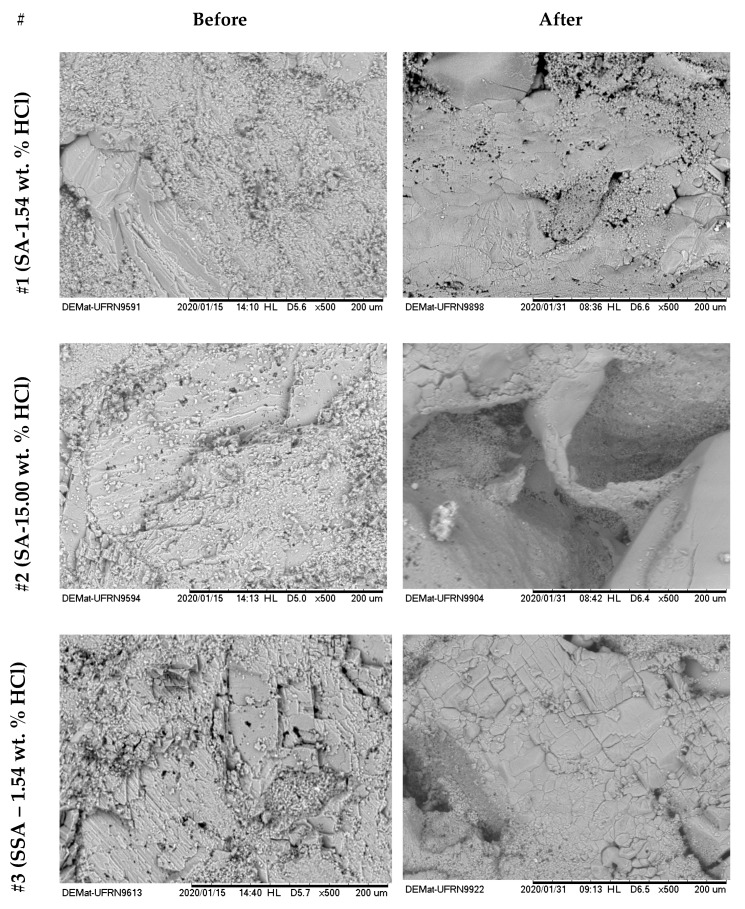
SEM photographs with 500× magnification in Indiana limestone, for systems SA-1.54 wt. % HCl (#1), SA-15.00 wt. % HCl (#2) and, SSA-1.54 wt. % HCl (#3), before and after contact angle measurement.

**Figure 17:**
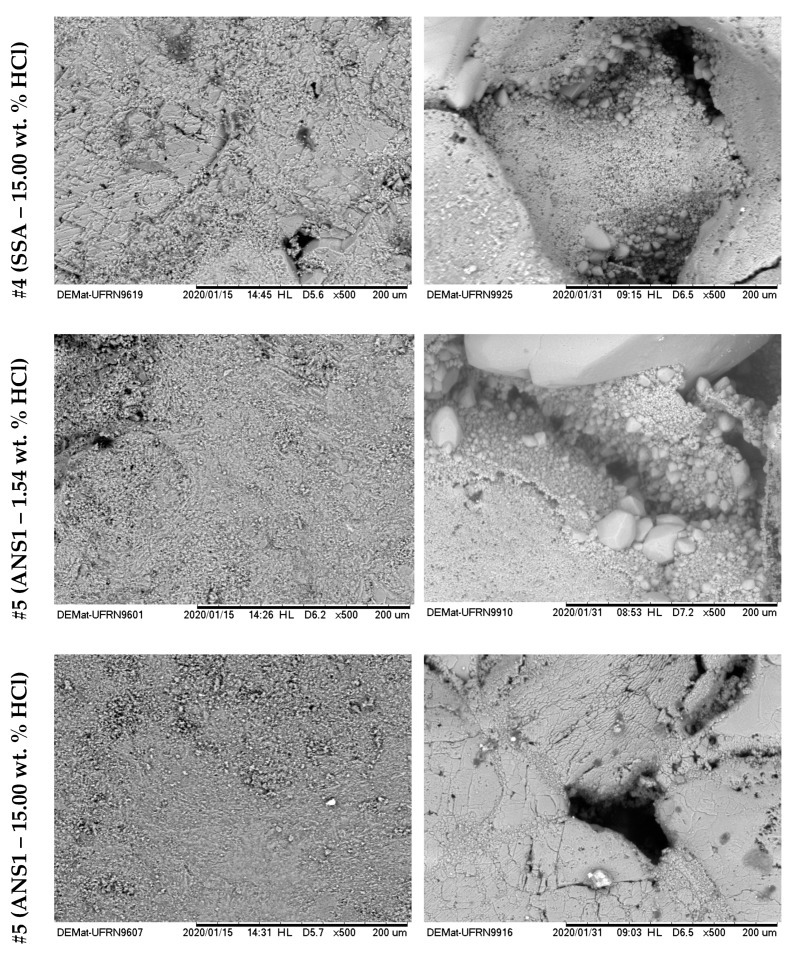
SEM photographs with 500× magnification for systems SSA-15.00 wt. % HCl (#4), ANS1-1.54 wt. % HCl (#5) and ANS1-15.00 wt. % HCl (#6), before and after contact angle measurement in Indiana limestone.

Figure 16 and Figure 17 show the surface images with 500× magnification for the six treatments reported in Table 4. The left side shows the images before the acid treatments, and the right side shows images after the respective acid treatment. 

From the analyses shown in Figure 16, it was possible to observe that for the systems #1 (SA–1.54 wt. % HCl) and #2 (SA–15.00 wt. % HCl), treatment systems were consumed on the surface or at a nearby point, a phenomenon similar to the dissolution on the face that occurs in rock plugs in low flow rates. This behavior occurs due to the high reactivity of HCl in an irrelevant limitation by mass transfer. It was observed that a deeper pit was formed for #2 compared with #1, due to the greater absolute availability of H+. 

For systems #3 (SSA–1.54 wt. % HCl), #4 (SSA–15.00 wt. % HCl), #5 (ANS1–1.54 wt. % HCl), and #6 (ANS1–15.00 wt. % HCl) (Figure 15 and Figure 16) it was possible to observe the formation of deep channels, beyond the detection limits of the equipment, compared to those obtained in experiments with aqueous systems #1 and #2. These deep channels were formed by the acid attack limited by the mass transfer that allowed the acid to reach areas further away from the surface, proving the retarding effect of media formed by auto-associative systems. The formation of these channels seemed to be associated with the behavior observed during the measurement of contact angle, in which, after intense spreading, the fluid disappeared on the surface.

Comparing the systems in the nanoemulsion media, #5 and #6, with the systems with surfactant in aqueous solution, #4 and #5, it was possible to observe a deeper penetration for the systems in nanoemulsion media. One explanation could be the greater retarding effect promoted by the droplets of nanoemulsion in relation to micelles only with surfactants.

## 3. Materials and Methods 

### 3.1. Materials

**Figure 18:**
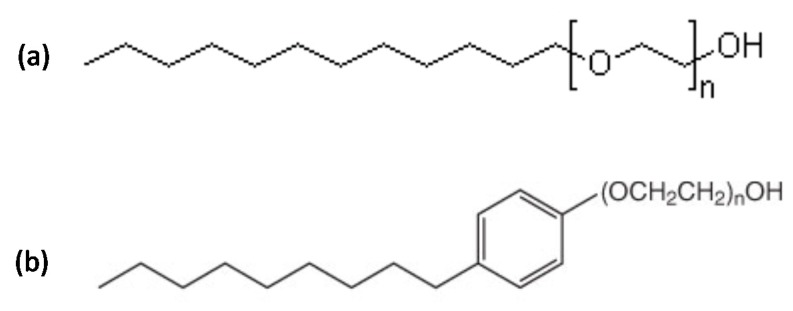
Surfactant molecular structures for (**a**) ALK100 and (**b**) RNX110 with “n” ethylene oxides.

ALK100 (lauryl alcohol with 10 ethylene oxides) and RNX110 (nonylphenol with 11 ethylene oxides) were the two non-ionic surfactants tested to obtain nanoemulsion systems with hydrophilic–lipophilic balances (HLB) of 13.9 and 13.7, respectively. Both were provided by Oxiteno S.A, São Paulo, BR, with a purity of >99 wt. %. Figure 18 shows the molecular structures for both surfactants, where “n” represents the average number of ethylene oxides present in the surfactant molecular structures. Butan-2-ol (Sec-butanol) was used as a co-surfactant (C), xylene was used as the oil phase (OP), and HCl aqueous solutions with different concentrations were used as the aqueous phase (AP). For the experiments of calcium carbonate dissolution, CaCO_3_ was used (Merkel, with a purity of >99 wt. %) in order to not consider other elements present in the rock formation and evaluate only the dissolution of the calcium carbonate.

### 3.2. Carbonate Coreflooding Experiments

**Figure 19:**
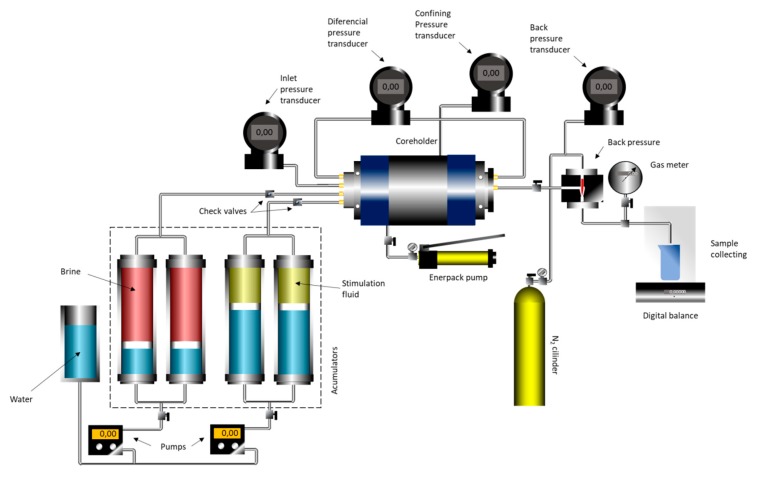
Schematic of coreflood setup.

Coreflooding experiments were performed to evaluate the efficiency of the acid nanoemulsions to stimulate carbonate formations and to form wormhole channels. Figure 19 shows the schematic of the experimental flow simulator used in the coreflood experiments. The experiments were performed at 30 °C with 2000 psi of overburden pressure in the core cell and back pressure at 1100 psi. All the experiments were performed using Indiana limestone plugs prepared with a diameter of 3.8 cm and a length of 5.0 cm. The plugs presented an average porosity of 15.4%. Injection of the acid systems into the core was performed at a constant volumetric pumping rate. Initially, the core sample was placed into the core holder, and the core’s initial pressure drop was measured using 2 wt. % KCl brine at three different flow rates, namely 0.5, 1.0, and 5.0 mL/min. A plot of flow rate versus pressure drop was then constructed. 

The slope was used to determine the permeability through the Darcy equation, given by Equation (1), where q is the volumetric flow rate (cm^3^/sec), d is the core diameter (cm), µ is the fluid viscosity (cP), and dP/dL is the pressure drop per unit length (atm/cm).

(1) $$k=-q\cdot \frac{4\cdot \mu }{\pi \cdot d^{2}}\cdot \frac{dL}{dP}$$

We then evaluated the effectiveness of the nanoemulsion systems to form wormhole channels at three different flow rates, namely 0.5, 1.0, and 5.0 mL/min. The injection was stopped when the differential pressure across the core reached a value near zero. This implies on the breakthrough of the wormhole. After the acidizing process, the core plugs were saturated with KCl 2 wt. %, and microCT imaging was processed to observe the wormhole formation pattern.

X-ray microtomography (microCT) imaging is a nondestructive method that allows internal structures in materials to be visualized. This method is widely used in various applications and has been used to scan carbonate and sandstone core plugs. An X-ray microCT scanner (inspeXio SMX-225CT, Shimadzu) was used to evaluate the wormhole formation. The core plugs were scanned at an applied X-ray tube voltage of 180 kV and a corresponding current of 70 μA. The source-to-object distance (SOD) was 29.4 mm, the source-to-image distance (SID) was 800 mm, the field of view in the xy plane (FOV(XY)) was 10 mm, the FOV in the z-direction (FOV(Z)) was 5 mm, and the voxel spacing was 0.010 mm/voxel.

### 3.3. Acid Nanoemulsion Preparation

Two acid nanoemulsion systems were obtained using ALK100 (ANS1) and RNX110 (ANS2) by the dilution of a microemulsion prepared in the water-in-oil (W/O) region. One used as a reference the microemulsion systems obtained by Aum et al. [13] with surfactant concentrations between 16.7 and 25.0 wt. %; in the present work, nanoemulsions with 5 wt. % of surfactant were obtained.

The change in concentration in order to obtain the nanoemulsion was classified as a low-energy emulsification method because it did not require elevated levels of energy input to form nanodroplets. The surfactant and the co-surfactant were mixed at a constant stirring rate (500 rpm) to obtain the nanoemulsion. The oil phase was then added. Subsequently, the aqueous phase was gently added while stirring the mixture at 500 rpm. Initially, the system formed was a water-in-oil (W/O) microemulsion. The continuous addition of acid solution caused a point shift to the oil-in-water (O/W) region of the microemulsion. Thus, by further addition of the aqueous phase, the system became an oil-in-water nanoemulsion. The acid systems were prepared at the temperature of 25 ± 1 °C. Surface tension was measured by maximum bubble pressure method, using a tensiometer (QC6000, SensaDyne Instruments, Milwaukee, WI, USA). In this method, the differential pressure is measured between two probes (capillaries immersed into the liquid) with different internal radii, where nitrogen (N_2_) is flowed through the probes, creating a bubble inside the liquid. The surface tension is obtained using the Young–Laplace equation [35,36,37]. Droplet diameter and polydispersity measures were performed using the method of dynamic light scattering (DLS) in a drop size analyzer (ZetaPlus Equipment, Brookhaven Instruments, Holtsville, NY, USA). The dynamic light scattering technique was used to study the droplet size and the stability of the nanoemulsions formed. The measurements were performed in five scans of 30 s each, using a detection angle of 15°, temperature of (30 ± 1 °C), laser of 40 mW with a wavelength of 640 nm, and precision of ± 3%.

Thermogravimetric analysis and visual phase equilibrium behavior under heating were used to evaluate the ANS1 and ANS2 systems’ thermal stability. First, samples of 5 mg of nanoemulsions were analyzed by thermogravimetric analysis (TGA-DTA), in which the masses of the systems were monitored as a function of temperature and time as they were subjected to a defined temperature program in the range of 300–700 K at a rate of 10 K/min in a controlled nitrogen atmosphere. The purge rate used was 50 mL/min. Subsequently, temperature stability experiments of 5 mL samples of nanoemulsions were carried out by monitoring changes in the phase equilibrium by visual observation during sample heating at 1 K/minute in an oil batch open to the atmosphere.

### 3.4. Rock Characterization

#### X-Ray Fluorescence (XRF) and X-Ray Diffraction (XRD)

X-ray fluorescence (XRF) was used to identify the elements and their quantities by the characteristic energy emitted of the transition between specific orbitals in an element [38]. Samples of the rocks were crushed and sieved on a 200 mesh screen, with about 1 g of the transfixing and homogenized material being analyzed. The Rh radiation used was between 15 and 50 kV in the Shimadzu spectrophotometer, model EDX 720. X-ray diffraction (XRD) is used for determining the geometric structures of a material, as well as helping to identify its composition. A specific set of peaks at determined angles are associated with specific compounds [39]. Therefore, samples of the Indiana limestone rocks were crushed and sieved on a 200 mesh screen, with about 1 g of the crushed homogenized material being analyzed. The equipment used was a Rigaku diffractometer, MiniFlex 2 model, at 3°/min, with steps of 0.01° and scan angles varying from 20 to 80° with Kα (Cu) radiation at 40 kV and 20 mA.

### 3.5. Dissolution of Calcium Carbonate in Nanoemulsion Media

To evaluate the effect on the reaction rate of the presence of dispersed nanodroplets in the fluid, the mass loss of calcium carbonate was qualitatively tested by registering the pressure in time during the reaction between the chloride acid and calcium carbonate in a confined cell under constant stirring. Pressure increased due to carbon dioxide (CO_2_) released as a product of the reaction shown in Equation (2).

(2) $$CaCO_{3}\;\left(s\right)\;+\;2\;H^{-}\;\left(l\right)\;\to \;Ca^{2+}\;\left(aq\right)\;+\;H_{2}O\left(l\right)\;+\;CO_{2}\left(g\right)\;↑$$

**Figure 20:**
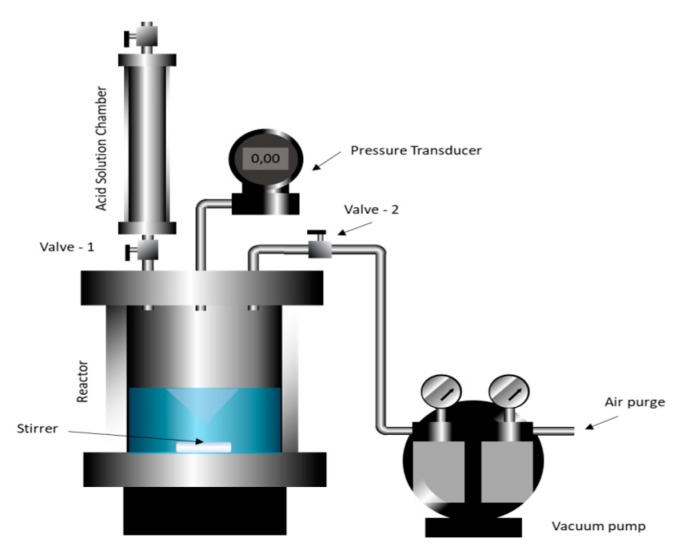
Reactor scheme.

The methodology used to evaluate the kinetics of the dissolution reaction was adapted from other works referenced [13,14,40]. Figure 20 shows the experimental equipment developed to perform the reaction and to measure the CO_2_ release. The initial mass of calcium carbonate m_0_ on the reaction vessel was 10 g of calcium carbonate in the reactor. The reactor was closed, and a low vacuum was created to allow the rapid acid release of the acid solution chamber. Next, the acid was released inside the reactor, and the reaction started. The pressure over time was registered using a digital manometer (MVR-87, Instrutherm, São Paulo, SP, BR) with an accuracy of ±1% (F.S.).

Pressure measurement was continued until no further pressure change was observed. The concentrations were calculated using Equation (3), where η is the percentage of calcium carbonate reacted, P is the pressure in the reaction cell, and Pmax is the pressure corresponding to the completing reaction of 10 g of calcium carbonate in excess HCl, i.e., the pressure of 100% conversion.

(3) $$\eta =\frac{P}{\mathrm{Pmax}}\;\cdot 100\%$$

### 3.6. Impact of Reactive Fluid–Rock Interaction on Rock Wettability

We evaluated the interactions between the Indiana limestone samples and HCl in (a) aqueous media, (b) with surfactant solution, and (c) in nanoemulsion media. 

The contact angle readings were performed using the sessile drop method with a goniometer KRUSS, model DSA100. The SEM experiments were carried out with a HITACHI, model TM3000, using 15 kV electron emission. The experiments were performed using slices of Indiana limestone with 38 mm diameter and 4.5 mm length.

## 4. Conclusions

In this study, a novel oil-in-water acid nanoemulsion fluid that can stimulate carbonate formations was experimentally demonstrated. The following conclusions could be drawn from the results.

- The surfactants used (RNX110 and ALK100) successfully formed liquid nanoemulsions through phase inversion promoted by dilution (low-energy method).
- The liquid nanoemulsions ANS1 and ANS2 presented great stability with time and temperature.
- It was possible to verify that the acid–carbonate reaction was retarded in the nanoemulsion media.
- After the reaction of the carbonate rock with the acid in surfactant solution and nanoemulsion media, the rock surface was released completely water wetted.
- SEM images demonstrated that the aqueous HCl tended to completely corrode the rock surface, while in nanoemulsion the formation of channels was verified, evidencing the retarded effect.
- Imaging experiments showed that the wormhole structures were formed by the injection of the liquid nanoemulsions. These findings indicated that the obtained nanoemulsions have the potential for application as retarded acid fluids in carbonate acidizing.

## Figures and Tables

**Table 1:** Coreflood experiment results.

#Run.	System	Plug Code	K_before_ (mD)	Porosity (%)	Flow Rate (mL/min)	PVBT
1	ANS1	IL-71D	7.2	14.5	0.5	3.0
2	ANS1	IL-67D	6.5	15.5	1.0	2.2
3	ANS1	IL-113	17.0	16.5	5.0	3.5
4	ANS2	IL-76D	7.0	14.7	0.5	2.7
5	ANS2	IL-72D	7.2	15.2	1.0	2.1
6	ANS2	IL-115	16.7	16.1	5.0	4.0

**Table 2:** Acid nanoemulsion systems composition.

System	S	C	AP	OP
HCL 15 wt. %			100 wt. %	
ANS1	5 wt. %	2.5 wt. %	91.5 wt. %	1 wt. %
ANS2	5 wt. %	2.5 wt. %	91.5 wt. %	1 wt. %

**Table 3:** Elementary compositional analysis for Indiana limestone.

Chemical Element	Ca	Fe	Si	S	K	Sr	Cu
%	97.63	1.15	0.20	0.08	0.26	0.37	0.31

**Table 4:** Resume of the reactive systems used in the study of wettability alteration.

Run	System Code	Continuous Media	HCl (wt. %)	Surfactant (wt. %)
#1	SA-1.54 wt. % HCl	Aqueous solution	1.54	0
#2	SA-15.00 wt. % HCl	Aqueous solution	15.00	0
#3	SSA-1.54 wt. % HCl	Surfactant solution	1.54	5
#4	SSA-15.00 wt. % HCl	Surfactant solution	15.00	5
#5	ANS1-1.54 wt. % HCl	Nanoemulsion (ANS1)	1.54	5
#6	ANS1-15.00 wt. % HCl	Nanoemulsion (ANS1)	15.00	5
